# The Role of Amygdala in Self-Conscious Emotions in a Patient With Acquired Bilateral Damage

**DOI:** 10.3389/fnins.2020.00677

**Published:** 2020-07-08

**Authors:** Luca Piretti, Edoardo Pappaianni, Alberta Lunardelli, Irene Zorzenon, Maja Ukmar, Valentina Pesavento, Raffaella Ida Rumiati, Remo Job, Alessandro Grecucci

**Affiliations:** ^1^Clinical and Affective Neuroscience Lab, Department of Psychology and Cognitive Sciences, University of Trento, Rovereto, Italy; ^2^Marica De Vincenzi Onlus Foundation, Rovereto, Italy; ^3^Rehabilitation Department, Ospedali Riuniti di Trieste, Trieste, Italy; ^4^Radiology Department, Ospedali Riuniti di Trieste, Trieste, Italy; ^5^Neuroscience and Society Lab, Neuroscience Area, International School for Advanced Studies (SISSA), Trieste, Italy

**Keywords:** amygdala, moral emotions, shame, emotion recognition, Guilt

## Abstract

Shame plays a fundamental role in the regulation of our social behavior. One intriguing question is whether amygdala might play a role in processing this emotion. In the present single-case study, we tested a patient with acquired damage of bilateral amygdalae and surrounding areas as well as healthy controls on shame processing and other social cognitive tasks. Results revealed that the patient’s subjective experience of shame, but not of guilt, was more reduced than in controls, only when social standards were violated, while it was not different than controls in case of moral violations. The impairment in discriminating between normal social situations and violations also emerged. Taken together, these findings suggest that the role of the amygdala in processing shame might reflect its relevance in resolving ambiguity and uncertainty, in order to correctly detect social violations and to generate shame feelings.

## Introduction

The amygdala is a subcortical nucleus which has been related to a broad variety of functions including facial emotion recognition, social cognition, and reward learning (Adolphs, 2010; Janak and Tye, 2015). While early findings highlighted the amygdala’s role in processing facial expressions, specifically fear expressions (Adolphs et al., 1994; Calder, 1996), and were initially interpreted by hypothesizing the amygdala involvement in processing stimuli signaling threat (Adolphs, 2010), more recent investigations highlighted that its role in processing faces might have something to do with the allocation of processing resources toward specific features to disambiguate facial expression meaning (Adolphs, 2010; Spezio et al., 2007). While patients with amygdala damage have been associated with reduced ability to recognize fearful faces (Adolphs et al., 1994; Calder, 1996), they, however, display spared abilities to recognize the same emotion by other body parts (i.e., gestures, Atkinson et al., 2007) or modalities (i.e., prosody, Adolphs and Tranel, 1999; Bach et al., 2013). Moreover, they also show reduced tendency to fixate the eye region (Adolphs et al., 2005; Spezio et al., 2007), ignoring the facial features that are diagnostic when recognizing fearful expressions (Smith et al., 2005). Aside from disambiguation, the amygdala might play further roles in emotional processing. Indeed, several neuroimaging studies reported that amygdala activation is sensitive to a wide repertoire of emotional stimuli, including both negatively and positively valenced items (Costafreda et al., 2008; Sabatinelli et al., 2011) and leading to the hypothesis that the amygdala might be involved in arousal processing (Anderson et al., 2003). Indeed, amygdala activation is modulated by the arousal of the stimuli (Anderson et al., 2003; Ball et al., 2009; Bonnet et al., 2015) and is coupled with psychophysiological responses (Bonnet et al., 2015).

Neuroimaging studies revealed that amygdala activation was associated with shame induction (Finger et al., 2006; Pulcu et al., 2014) and that amygdala volume correlated with shame proneness (Whittle et al., 2016). In addition, deep-brain stimulation of the amygdala induced in a patient the emotional experience of shame (Inman et al., 2018). Specifically, a patient’s emotional response was modulated by a stimulation intensity of 5 V associated with shame experience, and higher stimulations were associated with fear experience. In addition, these emotional responses were not evoked in other patients undergoing the same stimulation protocol (Inman et al., 2018). Together, these findings suggest that the amygdala might play a crucial role in generating shame experience.

However, other studies highlighted the amygdala’s role in understanding social situations (Martin and Weisberg, 2003; Noack et al., 2015; Lymer et al., 2018) and in detecting social violations (Berthoz et al., 2006; Bas-Hoogendam et al., 2017). These latter functions are highly correlated with shameful experiences, since shame generation requires accurate social situation assessment and is usually triggered by social and moral violations (Tangney et al., 2007). Specifically, shame usually occurs when an individual perceives the self or the persona as inadequate with respect to the accepted social and moral standards (Tangney et al., 1992), especially when a specific aspect of the self-image is perceived as defective (Gausel and Leach, 2011). In addition, shame generation leads to behavioral inhibition (Tangney et al., 2007) and, together with guilt, which is often associated with shame, promotes changing in the self and the behavior against immorality (Gausel and Brown, 2012; Martinez et al., 2014).

The aim of the present study is to clarify how amygdala damage can affect shame processing at different levels. In order to achieve this aim, we tested a patient with acquired brain damage at the level of bilateral amygdalae and surrounding tissues with different tasks tapping social cognitive skills, emotion facial recognition, and subjective experience. Specifically, we tested the subjective emotional experience of a patient by asking him to rate his level of shame associated with specific social situations. These situations involved violations of social and moral standards. If the amygdala is involved in generated emotional responses, we expect reduced shame emotional ratings compared with that in control participants in any condition. Conversely, if no difference or differences only in some conditions on the ratings between controls and the patient are present, it might not be attributed to primary emotional deficit. Hence, in this latter outcome, the role of the amygdala in moral judgment might not be ascribed to shame or guilt generation. Aside from this experimental task, to control for other basic deficits that might influence the outcome of this experimental tasks, the patient’s cognitive abilities and social cognitive skills were further investigated in a neuropsychological assessment. In addition, the patient was also evaluated on a set of emotion recognition tasks, following previous studies on patients with amygdala damage which reported deficits in recognizing fearful faces and spared ability to recognize emotion through body parts and prosody. Moreover, testing emotion recognition would allow us to ascertain whether shameful facial expression, which, different from other moral emotions, was also reported to be characterized by distinctive features (i.e., gaze movement downward and blushing) (Asendorpf, 1990; Keltner and Buswell, 1997), might also be impaired. Indeed, we hypothesized that shame experience deficit, if present, might also have impaired the patient’s ability to recognize the same emotion in others.

## Materials and Methods

### Case Description

**FIGURE 1:**
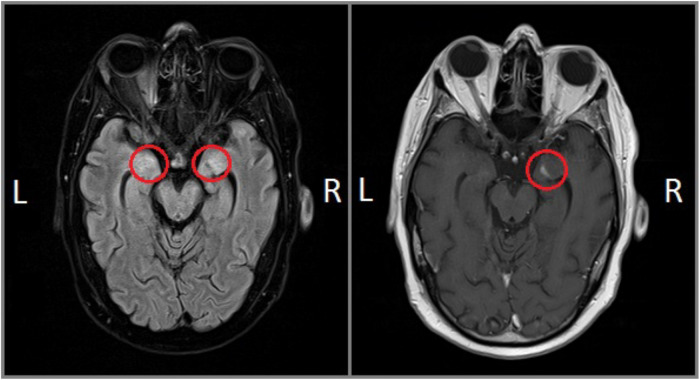
FF’s MRI scans: FLAIR **(left)** and T1-weighted gadolinium-enhanced **(right)** sequences.

FF is a right-handed middle-aged man, with 13 years of education, who was admitted to the rehabilitation ward with a diagnosis of Erdheim–Chester disease (non-Langerhans cells histiocytosis, see Diamond et al., 2014), with neurological and dermatological symptoms. About 2 years earlier, FF showed hyperprolactinemia and diabetes insipidus, and subsequently, he reported hyposthenia and hypoesthesia of the lower limbs, balance issues, emotional lability, and hypogeusia and received a diagnosis of gait ataxia and mild right hemiparesis. The MRI scan, acquired at the moment of the diagnosis, revealed bilateral cortical thickening mainly at the level of the amygdala. The lesion extended to the pituitary stalk, optic chiasm, and hypothalamus and involved also the lenticular nucleus, internal and external capsule in the left hemisphere, and the external capsule in the right hemisphere. Moreover, diffuse signal intensity alterations involved the cervical and thoracic spinal cord (mainly in the posterior columns). At the time of testing, the neurological symptoms had regressed, with a marked reduction of the emotional lability, mild improvement of the motor abilities, and minimal impairments in the cerebellar tests. Likewise, the MRI pattern at 5 months after the diagnosis and 4 months before the neuropsychological testing (see Figure 1) revealed a marked reduction in intensity alteration at the level of the amygdala, hippocampus, and pituitary stalk. Signal alterations located within the bilateral internal and external capsule, as well as at the level of right lenticular nucleus, were no longer detectable.

Before taking part in the experiment, patient FF, as well as a sample of healthy controls, signed an informed consent, which was approved by the local ethical committee (CEUR – regional ethical committee of Friuli Venezia Giulia). Healthy control samples included 13 age- and education-matched healthy male individuals (age: 48.6 ± 9.3, education: 13.8 ± 3.2, MMSE: 29.5 ± 0.5) who were tested on all the experimental tasks, as well as on Positive and Negative Affect Schedule (PANAS) and TOSCA. The sample size of the healthy control group was chosen based on previous single-case studies in the field of emotion processing (Pishnamazi et al., 2016; Bennetts et al., 2017; Bach et al., 2019).

For technical reasons, three individuals from the sample of the healthy controls were not tested in the emotion recognition from the prosody task and the emotional gestures recognition task, while another participant was not tested on PANAS.

### Neuropsychological Assessment

FF underwent a neuropsychological battery to assess his cognitive abilities (see Table 1). The battery included short- and long-term memory (prose memory), working memory (digit span backward and Corsi’s span backwards, attention (trail making test – part A) and executive functions (trail making test – part B, phonemic/semantic alternate fluency, WAIS similarities subtest, Tower of London test, Raven’s progressive matrices, Wisconsin card sorting test, verbal judgment test, and cognitive estimation test), fluency (phonological fluency and semantic fluency), praxis (freehand copying of drawings task and clock drawing test), and perception (facial recognition test). Finally, the PANAS (Crawford and Henry, 2004) was administered in order to assess the current affectivity of the participants. PANAS, consisting of 10 items measuring both negative and positive affect, is a self-report questionnaire in which participants are asked to indicate their level of experienced affect in that moment in a 5-point Likert scale.

**TABLE 1:** Neuropsychological battery and questionnaires.

Test	Range	Cutoffs	Raw score (corrected score)	*Z*-scores
**Memory**				
Digit span forward	0–9	< 4.26	6 (5.75)	
Corsi’s span forward	0–9	< 3.46	5 (4.74)	
Digit span backward	0–9	< 2.65	4 (3.71)	
Corsi’s span backwards	0–9	< 3.08	5 (4.77)	
Prose memory	0–28	< 7.5	14.5 (15)	
**Executive functions and attention**				
*Trail making test*				
-A	–	> 94	59 (56)	
-B	–	> 283	158 (152)	
Phonemic/semantic alternate fluency	–	< 12.7	26 (25.31)	
-Composite shifting index		< 0.38	1.15 (1.12)	
Similarities	0–28		6*	
Raven’s progressive matrices	0–36	≤ 18.96	34 (31.80)	
Tower of London test	0–36		32	-0.57^¥^
**Wisconsin Card Sorting Test**				
-Number of categories	0–6	≤ 2	2*	
-Number of perseverations	0–36	≥ 6.41	–	
Verbal judgment test	0–50	–	46 (40.25)	
Cognitive estimation test	0–27	≤ 12	9 (9.62)	
**Language**				
Phonological fluency	–	< 17.35	18 (13.3)*	
Semantic fluency	–	< 28.34	27 (27.34)*	
**Praxis**				
Freehand copying of drawings task	0–12	≤ 7.18	9 (8.4)	
Clock drawing test	0–10	≤ 8	8.5	
**Perception**				
Facial recognition test	0–54	< 39	39	
**Affective state**				
*PANAS*				
-Positive	0–50		37	0.95^§^
-Negative	0–50		18	-0.05^§^

*The table shows the patient’s performances on the neuropsychological battery including short-term (digit span forward and Corsi’s span forward, Monaco et al., 2013) and long-term memory (prose memory, Novelli et al., 1986), working memory (digit span backward and Corsi’s span backwards, Monaco et al., 2013), attention (trail making test – part A, Giovagnoli et al., 1996) and executive functions (trail making test – part B, Giovagnoli et al., 1996; phonemic/semantic alternate fluency, Costa et al., 2014; similarities subtest of WAIS, Wechsler, 2014; Tower of London test, Krikorian et al., 1994; Raven’s progressive matrices, Carlesimo et al., 1996; Wisconsin card sorting test, Caffarra et al., 2004; verbal judgment test, Spinnler and Tognoni, 1987; and cognitive estimation test, Scarpina et al., 2015), fluency (phonological fluency, Carlesimo et al., 1996; semantic fluency, Costa et al., 2014), praxis (freehand copying of drawings task, Carlesimo et al., 1996; clock drawing test, Mondini et al., 2003), and perception (facial recognition test, Benton et al., 1994; Albonico et al., 2017).* * *Impaired performance. ^§^ Obtained with healthy controls mean and standard deviation scores. ^¥^Obtained with normative data.*

### Social Cognition Battery

Social cognition battery (Prior et al., 2003) is a self-administered task with four different tests assessing different aspects of social cognition, namely, the emotion attribution, the theory of mind, the social situation, and the moral/conventional distinction. In each test, the participant is asked to read brief stories and to answer the related questions. In the emotion attribution test, brief stories describe one character in a specific situation (e.g., *Silvia wakes up and sees a poisonous spider in her bed*). The participant is asked to give a free answer to specific questions related to the feeling of the character (e.g., *How does Silvia feel in this situation?*). Stimuli include seven different emotions: sadness (*N* = 10), fear (*N* = 10), shame (*N* = 12), disgust (*N* = 3), happiness (*N* = 10), anger (*N* = 10), and envy (*N* = 3). In the theory-of-mind task, stories (*N* = 13) involved two or more characters interacting (e.g., *Katia and Emma are two children and are playing at home. Emma gets a banana and puts it close to her ear and says to Katia: Look, it’s a phone*). The participant must answer specific questions related to the character’s point of view (e.g., Is what Emma said true?). The social situation task includes stories in which two distinct social behaviors are highlighted (written in bold): one involves a normal social behavior and the other a social norm violation. The participant is asked to rate whether the behavior of the character can be considered normal, using the letters from “a” to “d” to indicate, respectively, a normal behavior to an extremely strange behavior. This test provides three scores: normal behavior identified, social violations identified, and the severity of the social violations. In the moral/conventional distinction test, stories related to children behaviors at school are presented. In half of the stories (*N* = 6), one character is a victim of harm or of an injustice by other characters (moral condition), while in the other half of the stories, one character is involved in a social rule violation, without provoking any injury to other individuals. Participants are asked to answer four questions: (1) whether the character is behaving in a proper way, (2) how serious is the behavior from a scale of 0 to 10, and (3) whether this behavior can be considered right in another country with different rules or (4) in case the teacher allows any children to behave as they want. Hence, for each condition of the moral/conventional distinction task, three different scores are provided: accuracy in detecting forbidden behavior (1), the severity of the violation (2), and the accuracy in detecting forbidden behavior without given rules (3 and 4).

### Shame and Guilt Task (SGT)

To measure participants’ subjective experience of shame and guilt, we developed the SGT. The SGT is a behavioral task that recreates several scenarios of social interaction between the participant and different partners. During this interaction, the participant is exposed to different social judgments concerning his person or behavior through verbal scripts. Such an interaction is recreated by proposing the partner’s photo in addition to the evaluation expressed in text form. To maximize the interpersonal aspect during such interactions, we have employed the face as a salient social stimulus, in addition to the assessment (our target stimulus).

Participants were asked to imagine that the person in the picture (the “judge”) expresses the judgment directed toward them, as in a real social interaction. In the test, stimuli included 18 pictures associated with 18 judgments. Judgments included two conditions: the “social standards” condition involved violations of social norms or social standards (e.g., “You have put on a lot of weight”) and the “harming others” condition involves injuries or harm toward an individual, made by the participant (e.g., “You destroyed my life”). While the social “standards condition” should elicit higher shame ratings, the “harming others” condition should elicit higher ratings of both shame and guilt, as proposed in previous studies (Lewis et al., 1993; Tangney et al., 2007). Pictures were taken from the NimStim database (Tottenham et al., 2009) and included Caucasian individuals of both genders (50% females). Participants were asked to imagine that the person in the picture (the “judge”) expresses the judgment directed toward them and to rate their subjective experience of shame and guilt on a Likert scale from 0 to 6.

### Emotion Recognition Tasks

#### Emotional Facial Expressions Recognition Task

In this task, we included 120 grayscale facial pictures taken from the Montreal Set of Facial Displays of Emotion (MSFDE, Beaupré et al., 2000). A subset of this database includes pictures obtained by morphing neutral and emotional pictures at various degrees (20, 40, 60, and 80%). We selected for each emotion (anger, disgust, fear, happiness, sadness, and shame) morphed pictures from 20 to 80% and fully emotional pictures. For each condition (each emotion at any intensity of expression), we included four items (i.e., four different face identities) (e.g., four trials for anger expressed with the intensity of 20%). Participants were asked to label the emotion presented into different labels (anger, disgust, fear, happiness, sadness, shame, and neutral).

#### Emotional Prosody Recognition Task

Participants were auditorily exposed to 48 sentences with neutral content (e.g., “the book is on the table”) and emotional prosody, through the use of headphones. The emotions were anger, fear, disgust, happiness, sadness, and surprise. Stimuli included four items for each emotion and were presented in random order. Participants were asked first to identify the emotion conveyed by the prosody by choosing among different options (anger, fear, disgust, happiness, sadness, surprise, and neutral) and then to rate the intensity of the emotion on a Likert scale from 0 to 7.

#### Emotional Gestures Recognition Task

The set of 32 grayscale body photographs expressing emotional body gestures used in the current task is derived from BEAST^1^ (De Gelder and Van den Stock, 2011; see also Cecchetto et al., 2014). The actors’ faces were covered by a gray circle, so that her/his facial emotion was not visible. Emotions included anger, fear, happiness, and sadness. Participants were asked to identify the emotion expressed by body gestures by selecting between five options – anger, fear, happiness, sadness, and neutral – and to rate on a Likert scale ranging from 1 to 7 the intensity of the emotion expressed.

### Statistical Analyses

The patient’s scores on the neuropsychological and social cognition batteries were compared with the available normative data, while those on the other tests, including PANAS, emotion recognition tasks, and SGT, were compared with the controls’ scores. Specifically, we used the software “SingleBayes_ES.exe^1^,” implementing the method described by Crawford and Garthwaite (2007) and Crawford et al. (2010), which is widely used in case report studies and allows controlling for type I errors when comparing the patient’s and controls’ performance scores. This method estimates, within a Bayesian framework, the point of abnormality of the patient’s score (PA) and the associated 95% credible limits (CL). In addition, the PA provides the percentage of the healthy population obtaining a score lower than the patient’s. Then, in case of deficit, a second analysis was performed (e.g., Bayesian standardized difference test) (Crawford and Garthwaite, 2007; Crawford et al., 2010), using the software DissocsBayes_ES.exe^2^, to test whether the patient’s performance reduction is significantly lower than other scores of the same task, configuring a strong or classical dissociation (Crawford and Garthwaite, 2005). Since we expected that FF was impaired in these tasks, one-tailed tests were used. This method of analysis was applied to PANAS, emotion recognition from a prosody task, the emotional gestures recognition task, and SGT.

The facial emotion recognition task was first analyzed with one-sample *t*-tests (one-tailed) vs. chance level with the software Jamovi^3^ to test whether participants performed above chance level at any intensity of emotional expressions. Secondly, the patient’s and controls’ performances were compared. Since participants were asked to choose between six options in the task (anger, disgust, fear, joy, sadness, and shame) in four trials for each emotional intensity, the chance level was set to 0.07. The patient’s performances on this task were considered at chance level if their total accuracies for each emotional intensity were equal to 0 or 1, while they were considered above chance if the score was 2, 3, or 4. Then, given the complexity of the design, the patient’s and controls’ performances on facial emotion recognition task were compared with mixed-effect models (MMs), using the program R^4^ and the package lme4^5^. MMs represent a powerful tool in the analysis of single-case data, allowing us to compare the patient’s and controls’ performances even in complex study designs, such as repeated-measure designs (Huber et al., 2015; Wiley and Rapp, 2018). Specifically, we used a generalized mixed-effect model (function glmer) on the accuracy of the facial emotion recognition task (binomial) using the subject and the identity of the actor in the stimuli as random factors and the group (patient and controls), the emotion type (anger, disgust, fear, joy, sadness, and shame), the emotion intensity, and their interactions as fixed factors. Then, we removed stepwise any fixed factors not inducing any significant loss of fit to the model (tested with the likelihood ratio test). The final model included as fixed factors the interaction between group and emotion and the interaction between emotion and intensity. To explore the interactions, we performed a planned contrast between the patient’s and controls’ scores for each emotion type (*lsmeans*^6^). Then, similar to the analyses of the other tasks, in case of deficit, we tested whether the patient’s performance reduction on one emotion was significantly different from those of other emotions. Specifically, we contrasted the difference in the patient’s and controls’ performances on each impaired emotion and those related to other emotions. Bonferroni corrections were also applied.

## Results

### Neuropsychological Assessment and Questionnaires

FF’s results are summarized in Table 1. He was impaired at the Wisconsin’s card sorting test and similarities subtest of the WAIS. battery, suggesting a deficit affecting abstraction abilities. His performances on phonological and semantic fluencies were also poor, while on alternate semantic/phonological fluency, it was at the average level. The patient’s score on Benton’s facial recognition test was in the borderline range. The affective state of FF, measured by the positive and negative scores of PANAS, was not different from that of healthy controls [positive affect score: FF = 37, controls = 29.92 ± 7.43, *Z* = 0.95, PA = 81.17 (60.92–94.51), *p* > 0.1; negative affect score: FF = 18; controls = 18.33 ± 7.28, *Z* = −0.05, PA = 48.30 (27.84–69.16), *p*s > 0.1].

### Social Cognition Battery

The emotion attribution task (see Table 2) revealed that FF was impaired in attributing sadness and disgust to characters of brief stories, while his performance on fear, shame, happiness, anger, and envy was above the cutoff. It is worth noting that errors in sadness and disgust attributions included mainly anger (errors on sadness: 80% anger, 20% shame; errors on disgust: 100% anger). On the social situation task, FF showed impaired abilities in identifying normal social behavior and social violations, while his evaluation of the severity of the social violation was just above the cutoff. The patient’s performances on both the theory-of-mind task and the moral/conventional distinction task were in normal ranges.

**TABLE 2:** Patient’s scores on the social cognition battery.

Test	Score	Range	Cutoffs
Theory of mind	**12**	**0–13**	**≥ 12**
**Emotion attribution**			
-Sadness	5*	0–10	≥ 6
-Fear	10	0–10	≥ 8
-Shame	10	0–12	≥ 8
-Disgust	1*	0–3	≥ 2
-Joy	9	0–10	≥ 10
-Anger	9	0–10	≥ 6
-Envy	3	0–3	≥ 1
**Social situations**			
-Identification of correct social behaviors	12*	0–15	≥ 13
-Identification of social violations	20*	0–25	≥ 22
-Rating of the entity of violations	45	0–75	≥ 45
**Moral/conventional distinction**			
-Moral behaviors	6	0–6	≥ 6
-Conventional behaviors	6	0–6	≥ 5

* Impaired performance.

### SGT

**FIGURE 2:**
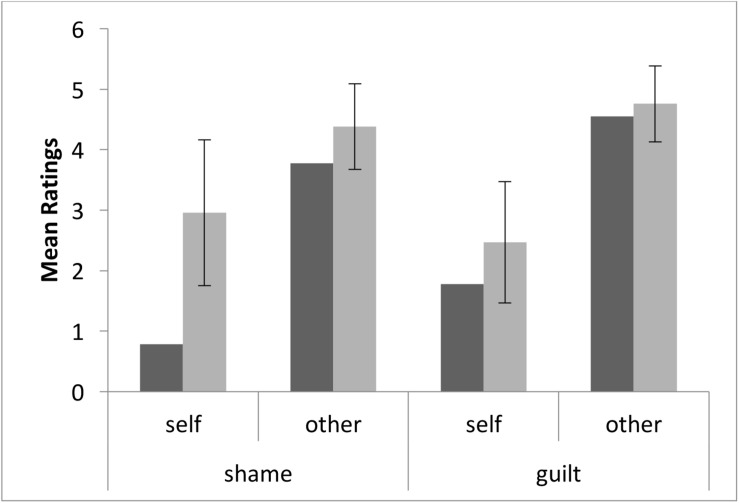
Patient’s (dark gray) and healthy controls’ (light gray) scores on the SGT. The error bars indicate standard deviations.

FF’s shame ratings (see Figure 2) on the “social standards” condition were marginally lower than those of healthy controls (FF = 0.78, healthy controls = 2.96 ± 1.20, Zcc = −1.82, PA = 5.28, superior CL = 15.00, *p* = 0.053), and his ratings on the “harming others” condition were within the control level (FF = 3.78, healthy controls = 4.38 ± 0.71, Zcc = −0.85, PA = 21.58, superior CL = 38.44, *p* > 0.1). Moreover, patient’s ratings of guilt were not different from those of controls in any condition (social standards: FF = 1.78, healthy controls = 2.47 ± 1.00, Zcc = −0.69, PA = 25.95, superior CL = 43.41, *p* > 0.1; harming others: FF = 4.56, healthy controls = 4.76 ± 0.63, Zcc = −0.32, PA = 38.25, superior CL = 56.27, *p* > 0.1. The reduction in shame ratings for the “social standard” condition was also significantly different than guilt ratings on the same condition (Z-dcc = −1.90, PA = 4.65, superior CL = 20.41, *p* < 0.05), while it was not significantly different than shame ratings on the “harming others” condition (Z-dcc = −1.03, PA = 17.12, superior CL = 44.55, *p* > 0.1).

### Facial Emotion Recognition Task

**FIGURE 3:**
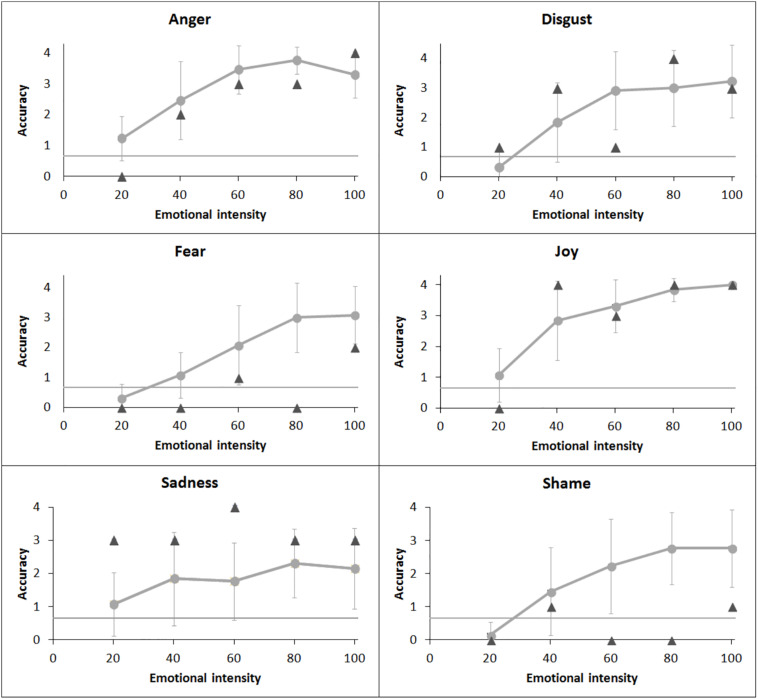
Patient’s (dark gray triangle) performance and healthy controls’ (light gray dot) mean performance on emotional facial recognition task. Bars indicate standard deviations.

Healthy individuals recognized all the emotions above chance level when the intensities ranged between 40 and 100% (all *p*s < 0.05). When the intensity was 20%, controls’ performances on anger were also above chance level (*p* < 0.01), but this was not the case for all the other emotions (all *p*s > 0.05) (see Figure 3 and Table 3). Patient FF showed a similar pattern to that of healthy controls when emotions were expressed at the 20% intensity, except for sadness, which was recognized above chance level, and for disgust at 60%, which was recognized at chance level. In addition, FF recognized shameful facial expressions at chance level at any intensity of presentation, while he performed at chance level when fear was expressed at 40, 60, and 80% intensities.

**TABLE 3:** FF’s and controls’ performance in the Facial Emotion Recognition Task for all intensity levels.

Emotion	Intensity														
	
	20%			40%			60%			80%			100%		
					
	HC	FF	Zcc	HC	FF	Zcc	HC	FF	Zcc	HC	FF	Zcc	HC	FF	Zcc
Anger	1.23 ± 0.73	0	–1.7	2.46 ± 1.27	2	–0.36	3.46 ± 0.78	3	–0.59	3.77 ± 0.44	3	–1.75	3.31 ± 0.75	4	0.92
Disgust	0.31 ± 0.63	1	1.1	1.85 ± 1.34	3	0.86	2.92 ± 1.32	1	–1.46	3.00 ± 1.29	4	0.77	3.23 ± 1.24	3	–0.19
Fear	0.31 ± 0.48	0	–0.64	1.08 ± 0.76	0	–1.42	2.08 ± 1.32	1	–0.82	3.00 ± 1.15	0	–2.6	3.08 ± 0.95	2	–1.13
Joy	1.08 ± 0.86	0	–1.25	2.85 ± 1.28	4	0.9	3.31 ± 0.85	3	–0.36	3.85 ± 0.38	4	0.41	4.00 ± 0.00	4	/
Sadness	1.08 ± 0.95	3	2.02	1.85 ± 1.41	3	0.82	1.77 ± 1.17	4	1.91	2.31 ± 1.03	3	0.67	2.15 ± 1.21	3	0.70
Shame	0.15 ± 0.38	0	–0.41	1.46 ± 1.33	1	–0.35	2.23 ± 1.42	0	–1.57	2.77 ± 1.09	0	–2.54	2.77 ± 1.17	1	–1.52

The mixed-effect generalized linear model (logLik = −854.2, marginal *r*^2^ = 0.39, conditional *r*^2^ = 0.44) revealed a significant main effect of emotion [*χ*^2^(5) = 48.18, *p* < 0.001] and intensity [*χ*^2^(1) = 232.85, *p* < 0.001] and significant interactions of group ^∗^ emotion [*χ*^2^(5) = 27.34, *p* < 0.001] and emotion ^∗^ intensity [*χ*^2^(5) = 49.21, *p* < 0.001]. Participants were overall more accurate in recognizing faces displaying joy than all other emotions (joy vs. sadness: *z* = 2.71, *p* = 0.07, all other *p*s < 0.05), except for those displaying anger (*p* > 0.1). Shame and fear were recognized less accurately than all the other emotions (all *p*s < 0.05). However, no significant difference was evident from the comparisons between FF’s and controls’ performances for any emotion displayed (all *p*s > 0.05). Indeed, even though FF recognized shame and fear at chance level, while controls performed above chance, the difference among their performances did not reach significance level (fear: *z* = −2.43, *p* = 0.088; shame: *z* = 2.55, *p* = 0.063).

### Emotional Gestures Recognition Task

FF’s performance on emotional gesture recognition task (see Table 4) did not differ from that of healthy controls in any of the emotions investigated (all *p*s > 0.01), highlighting that the patient was not impaired in recognizing emotions from body gestures.

**TABLE 4:** FF’s and controls’ accuracy scores in the different emotion recognition tasks.

	FF	Controls		Zcc	PA (Upper CL)
		
		*Mean*	*SD*		
**Body emotion recognition accuracy**					
Anger	6	6.90	1.60	–0.56	30.25 (54.87)
Fear	8	7.40	0.70	0.86	78.26 (92.68)
Joy	3	5.10	2.13	–0.99	18.60 (37.39)
Sadness	8	8.00	0.00	–	–
**Prosody emotion recognition accuracy**					
Anger	4	3.50	0.71	0.70	74.06 (89.85)
Disgust	3	1.90	1.20	0.92	79.76 (93.62)
Fear	4	3.40	0.97	0.62	71.51 (88.00)
Joy	3	3.10	0.99	–0.10	46.28 (66.41)
Sadness	3	3.50	0.53	–0.94	19.60 (30.64)
Surprise	3	3.00	0.94	0.00	50.01 (69.86)

**Zcc, effect size; PA, point of abnormality of the patient’s score, expressing the percentage of the healthy population falling below FF’s score.**

### Emotion Recognition From Prosody

The analyses of emotion recognition of auditory stimuli did not show any significant difference in patient’s and controls’ performances (all *p*s > 0.1) (see Table 4).

## Discussion

In the present single-case study, we tested the role of the amygdala in the perceptual and experiential processing of shame. Patient FF, with acquired bilateral amygdala and hippocampal damage, performed several tests tapping subjective emotional experience of shame, emotion recognition, and social cognition.

The assessment of the subjective experience of shame revealed two different patterns of findings. FF experienced less shame than controls when exposed to social standard violations but not to moral violations. FF’s and controls’ guilt ratings did not differ in any condition. This pattern of results is not congruent with the view of a primary role for the amygdala in shame generation. Indeed, if the amygdala was involved in the generation of a subjective experience of shame, after its lesion, we would expect a reduction of both shame ratings across all situations and not only in association with social standard violations.

However, the selective reduction in FF’s shame experience in reaction to social standard violation might be easily explained considering his deficit in recognizing whether a social situation was normal or not in the social cognition battery. Indeed, FF’s reduction in the subjective experience of shame might be secondary to the impaired ability to detect whether a social situation is to be considered normal: if an individual is not able to detect the occurrence of a social violation, she will not be able to react properly to such violation. This latter finding might be interpreted at least in two ways. First, the patient lacks social knowledge and, hence, is not able to compare the perceived social stimuli to prior knowledge, and second, he is not able to detect the relevant cues that are necessary for understanding the social situation and that need to be matched with prior social knowledge. While several studies reveal that the crucial region involved in representing social knowledge is the anterior temporal lobe (Olson et al., 2013; Wang et al., 2017) and that a lesion of this area, even sparing the amygdala, leads to pervasive impairments in emotional and social behaviors (i.e., psychic blindness) due to degraded social knowledge (Franzen and Myers, 1973), the amygdala was proposed to be involved in disambiguation, orienting attention to salient cues in order to understand stimulus meaning (Whalen, 1999; Adolphs, 2010). Indeed, neuroimaging studies revealed that amygdala activation was modulated by the ambiguity of the stimulus (Davis et al., 2016; Wang et al., 2017). FF’s ability to discriminate between moral and conventional social situations was spared, possibly because the situations presented were less ambiguous, and this is consistent with our interpretation that FF suffers from deficit in disambiguating the stimuli. Even though it is not possible to exclude that the patient’s impairment in detecting social violations might be due to the involvement of structures in close proximity to the amygdala, we argue that the role of the amygdala in orienting attention to salient cues to deal with ambiguous social situations might explain this deficit.

In addition, FF also showed impaired performances on some tasks tapping executive functions such as the similarity task, Wisconsin card sorting test, and verbal fluency tasks. However, FF’s deficit was not extended to all the tests tapping executive functions, but only to those requiring abstraction abilities. Recent evidence (Saez et al., 2015) suggested not only that frontal lobes are crucial for executive functions (Alvarez and Emory, 2006) but also that the amygdala might contribute to high-order cognitive functions, specifically being involved in representing abstract cognitive information. This interpretation of FF’s performance on neuropsychological battery might also better explain the patient’s deficit in understanding social situations, which are abstract in nature.

Results on the emotion recognition task revealed that, even though there was no significant difference between the performances of FF and controls, the patient recognized shameful and fearful facial expressions at chance level, while controls performed above chance. This might be attributable to the low number of trials per condition and, consequently, to the low sensitivity of this task to detect mild deficits. However, the inability to recognize fearful and shameful facial expressions of FF, although not different from that of controls, might reflect nevertheless a deficit in recognizing the two emotions. While the deficit at recognizing fearful facial expressions is consistent with previous evidence (Adolphs et al., 1994; Calder, 1996), shameful facial expression recognition has not been systematically investigated before. Social emotion recognition impairment (including moral emotions) from faces was only reported in patients with acquired amygdala damage (Adolphs et al., 2002), without any distinction about the specific facial emotion impaired. The same emotions were not impaired in the emotion attribution task of the social cognition battery, indicating that FF was able to recognize shame and fear from written stories, but he was impaired when faces were used as stimuli. In addition, FF’s ability to recognize specific emotions from bodily gestures and from prosody was completely preserved. FF’s poor performance on fear recognition, which was limited to facial expression, not involving bodily gestures and prosody, confirms previous research on patients with amygdala damage (Adolphs and Tranel, 1999; Atkinson et al., 2007; Bach et al., 2013). This pattern of results can be explained with the amygdala being involved in orienting attention to the eye region when presented with a facial stimulus (Jacobs et al., 2012). Indeed, the eye region is diagnostic in the identification of fear (Smith et al., 2005) and is poorly fixated by patients with amygdala damage during face presentation (Adolphs et al., 2005; Spezio et al., 2007).

Although whether the eye region might be diagnostic of shameful expression recognition has never been tested, the action tendencies associated with the shame experience seem to involve gaze movement downward, blushing, and inhibition of speech and movement (Asendorpf, 1990; Keltner and Buswell, 1997). Hence, the deficit of allocating attention toward the eye region might prevent patients with amygdala damage from perceiving a shift of gaze direction downward, typically associated with shameful facial expression.

Different from facial expression recognition, FF performed poorly in sadness and disgust attribution from brief stories on the social cognition battery. Previous studies highlighted the association between disgust and sadness, and amygdala and hippocampus processing. Indeed, a recent study reported that increased variability of a subnetwork formed by the amygdala and the hippocampus correlated with worsening mood and depression (Kirkby et al., 2018). For disgust, a recent study by Pujol et al. (2018) found the involvement of the hippocampus in response to disgusting food, and another experiment (Blanco-Hinojo et al., 2019) showed abnormal responses to disgusting food inside the hippocampus of individuals affected by Prader–Willi syndrome when compared to controls. We might speculate that the patient might have performed poorly in detecting sad and disgusting scenes in the attribution task because a damage of the hippocampus (for disgust) and of the amygdala–hippocampus circuit (for sadness) could have led also to mild deficits in processing sad and disgusting scenes. However, further research is necessary to confirm our findings.

## Conclusion

The investigation of shame and guilt processing in a patient with acquired damage within bilateral amygdalae and surrounding tissues revealed reduced feelings of shame in self-relevant social situations in association with a deficit in discriminating normal social situations and social violations and impaired performance on the Wisconsin card sorting test and WAIS analogies. In addition, the patient performed poorly in shameful (and fearful) facial expression recognition. This pattern of findings is congruent with a deficit in detecting salient cues in order to understand social situations and, consequently, to generate shame feelings in case of violations. Hence, the amygdala integrity appears to be relevant in the detection of social stimuli but not in the generation of moral emotions such as shame and guilt. These findings are more easily explained assuming a role of the amygdala in ambiguity and uncertainty resolution, as suggested by Whalen (1999). However, further research is necessary in order to better understand the role of the amygdala in moral emotion processing.

### Limitations

The present study involves the testing of a patient with a bilateral lesion of the amygdala that extends to the surrounding part of the hippocampus. Hence, the reported deficits might also be attributable to the lesion of both the hippocampus and the amygdala. Moreover, MRI acquisition and cognitive testing occurred at different time points (i.e., 4 months’ interval). Hence, the patient’s behavioral findings might not correspond strictly to the detected damaged brain areas.

The lack of data about premorbid patient’s cognitive performances does not allow us to make causal inferences about the role of the damaged areas in influencing behavior. However, the associations between specific brain lesions and impaired behavioral performances give interesting hints on the role of amygdala. In addition, the findings of single-case studies have low generalizability and need to be confirmed by further group studies. However, the relative rarity of the case described gives an important contribution in the understanding of amygdala functioning.

## Data Availability Statement

The datasets generated for this study are available on request to the corresponding author.

## Ethics Statement

The studies involving human participants were reviewed and approved by the Comitato Etico Unico Regionale (CEUR), Friuli-Venezia Giulia. The patients/participants provided their written informed consent to participate in this study. Written informed consent was obtained from the individual(s) for the publication of any potentially identifiable images or data included in this manuscript.

## Author Contributions

LP collected the data of the experimental tasks on the patient, analyzed the data, and drafted the manuscript. EP collected the data on the part of the healthy control samples and revised the manuscript. AL collected the neuropsychological data on the patient. MU and IZ interpreted the radiological findings. EP, VP, RR, RJ, and AG revised the manuscript. LP and AG interpreted the results. AG designed and supervised the project. All authors contributed to the article and approved the submitted version.

## Conflict of Interest

The authors declare that the research was conducted in the absence of any commercial or financial relationships that could be construed as a potential conflict of interest.

## Footnotes

1 www.beatricedegelder.com/

2 https://homepages.abdn.ac.uk/j.crawford/pages/dept/psychom.htm

3 https://www.jamovi.org/

4 https://www.r-project.org/

5 https://cran.r-project.org/web/packages/lme4/

6 https://cran.r-project.org/web/packages/lsmeans/index.html
